# Anticipatory and Compensatory Postural Adjustments in Sitting and Standing Positions During Functional Activities in Children With Cerebral Palsy

**DOI:** 10.1002/pri.70028

**Published:** 2025-01-13

**Authors:** Priyal Vasani, Amitesh Narayan, Akshatha Nayak, Mohammed Alsulaimani, Abdul Rahman Alzahrani

**Affiliations:** ^1^ Department of Physiotherapy Kasturba Medical College Mangalore, Manipal Academy of Higher Education Manipal Karnataka India; ^2^ King Fahad Armed Forces Hospital Jeddah Saudi Arabia

**Keywords:** anticipatory postural adjustment, cerebral palsy, compensatory postural adjustment, postural control

## Abstract

**Background and purpose:**

Anticipatory postural adjustments (APA) and compensatory postural adjustments (CPA) have played a vital role in postural control since early childhood, which is critical to accomplishing activities in daily life. However, literature indicated dissimilarities and inconsistencies in APA and CPA analysis in sitting and standing positions in children with Cerebral Palsy (CP). Thus, this study analyzed the changes in postural control (APA and CPA) through the postural muscles [rectus abdominis (RA) and erector spinae (ES)] in both standing and sitting positions during functional activities (grasping a ball) in children with CP.

**Methods:**

Children with CP [*N* = 21] aged 5–13 years having GMFCS levels I (*n* = 12) and II (*n* = 9) participated. Surface electromyography (EMG) was performed for postural muscles (ES and RA) to measure the APA and CPA with two types of loads (heavy and light) in both sitting and standing positions.

**Results:**

Children with CP showed increased EMG amplitude for APA and CPA with a heavier load than light load in sitting and standing positions. The EMG amplitude of CPA in sitting and standing for both load conditions was significantly higher than that of APA.

**Discussion:**

The findings suggest rehabilitation programs should enhance APA and CPA through targeted exercises and load management strategies. These insights have the potential to inform clinical practices, improve postural stability, and ultimately strengthen the ability of children with CP to perform daily activities with greater ease and confidence, thereby significantly impacting the quality of life.

**Abbreviations**
APA: Anticipatory postural adjustment
COP: Center of pressure
CP: Cerebral palsy
CPA: Compensatory postural adjustment
EMG: Electromyography
ES: Erector Spinae
MVC: Maximum voluntary contraction
RA: Rectus abdominis
SBCP: Spastic bilateral cerebral palsy
TD: Typically developing children
μV: Micro Volt

## 1 Introduction

The deficient postural control is the primary concern in children with CP (Petersen, Kube, and Palmer 1998), which is important for executing functional activities, that is, gross/fine motor skills (locomotion, prehension skills, etc) (Pierret et al. 2021). The trunk is the key frame of reference to maintain postural balance during voluntary activities (Van Der Heide et al. 2004). These voluntary movements need postural adjustment strategies, that is, Anticipatory postural adjustments (APA) and compensatory postural adjustments (CPA) (Massion 1994, 1998). The APA is seen in typically developing (TD) children from the age of 15 months during reaching while seated, which is used as a proactive maneuver to correct balance disturbances in daily activities (Van der Fits et al. 1999). Research in infants shows that CPA in standing develops between 10 and 12 months of age (Westcott et al. 2004). When balance is disturbed by voluntary movements, postural muscles are activated before the onset of the movement to perform the movement adequately. The ability to adequately generate this type of postural control, known as APA, is essential for performing various voluntary movements while maintaining posture and equilibrium. After the perturbation has occurred, sensory feedback signals are initiated to restore balance, which is executed by the CPA (Massion 1992; Toussaint et al. 1998).

Postural control is a prerequisite for many daily activities, but studies have reported that it develops differently in children with CP. Several changes occur in the postural control of children with CP due to an imbalance between the activation of ventral (RA) and dorsal (ES) postural muscles. Some higher functioning children with CP, that is, GMFCS level I and II, can stand and walk independently with or without assistive devices even though deficits in postural control persist. The exact nature of the deficits is only known to a limited extent. These deficits are a lack of direction‐specific postural adjustments and an inability to adjust postural activity to task‐specific modulations (Woollacott et al. 1998; Rose et al. 2002).

Available studies suggest that many approaches, that is, EMG, kinematic, and kinetic measures of the center of pressure (COP), have been used to examine postural control (Stapley, Pozzo, and Grishin 1998; Riach et al. 1990). EMG is the most common measure to document the anticipatory bursts of muscle activity. The amplitude of the EMG signals indicates the magnitude of muscle activity, the number of active motor units, and the activation frequency (Berg et al. 2012).

Children with CP showed large variations in trunk muscle activation during perturbation. Most APA studies involving children examined the anticipatory changes in the COP before forward or backward arm reaching movements in a standing position. A study that examined the association of anticipatory postural activity (APA) during bilateral arm flexion in standing among children with spastic diplegia found that the postural muscles, that is, ES, were activated before focal muscles (anterior deltoid) with a reduced onset of burst by 0.7%–12% indicating inadequate postural muscle activity. This study did not examine the CPA (Tomita et al. 2010).

In a comparative study, APA was examined during bilateral arm flexion and extension in standing between spastic hemiplegia and spastic diplegia with TD. They found greater APA activity in the ES muscles for the TD group than for the hemiplegic and diplegics during shoulder extension, while TD and hemiplegics demonstrated larger APA activity in the RA muscle. They also reported significantly higher baseline EMG amplitude for ES muscle among diplegics than the TD and hemiplegics, indicating the increased preactivated state of ES muscles (Girolami, Shiratori, and Aruin 2011).

Another comparative study examined the APA and CPA in a sitting position involving children with CP and TD using heavy and light loads. Results revealed that the EMG amplitude of anterior trunk muscles (RA) during APA was reduced compared to the resting level for both ball conditions among children with CP. Additionally, the intensity of EMG for the trunk muscles (RA and ES) was higher for the heavy ball than the light ball condition during APA (44.9 ± 20 and 19.0 ± 9.6, respectively). Conversely, during CPA, the EMG amplitude was higher for the light ball than the heavy ball (58.2 ± 18 and 56.9 ± 17, respectively). Overall, the EMG amplitude was significantly higher during CPA compared to APA for TD and children with CP (Bigongiari et al. 2011).

The evidence suggests dissimilarities and inconsistencies concerning APA and CPA analysis in sitting and standing positions in children with CP. The potential reasons could be (1) inclusion of variable GMFCS levels (I, II, and III) (Tomita et al. 2010; Girolami, Shiratori, and Aruin 2011; Bigongiari et al. 2011); (2) group analysis of children with CP without topographical differentiation; and (3) selection of either sitting (Van Der Heide et al. 2004; Bigongiari et al. 2011; van der Heide et al. 2003) or standing position in all the studies (Tomita et al. 2011; Girolami, Shiratori, and Aruin 2010).

Children with GMFCS levels I and II have near‐normal functions and can engage in more autonomous activities. Hence, they were chosen for the study.

Therefore, this study assessed the change in APA and CPA in standing and sitting positions during functional activities in children with CP having motor functional levels I and II on GMFCS.

## 2 Methods

### 2.1 Study Population

This prospective study was conducted between February 2023 and March 2024 at the Neurosensory Development Unit of a tertiary hospital associated with the medical college. This study was approved by the Institutional Ethics Committee (IEC) and the Scientific Committee (IECKMCMLR‐01/2023/18), and it meets the ethical principal guidelines of the Declaration of Helsinki. Written informed consent was obtained from the parents/caregivers of the participating children.

Children with a medical diagnosis of CP, aged 5–13 years, were included. Only children who could stand and ambulate independently, that is, GMFCS levels I and II, were included. Exclusion criteria were history of musculoskeletal or neurological surgery, receipt of antiepileptics or anti‐spastic medications or neurotoxin injections in the past 6 months, or if the child was cognitively challenged or non‐cooperative.

### 2.2 Procedures

**FIGURE 1:**
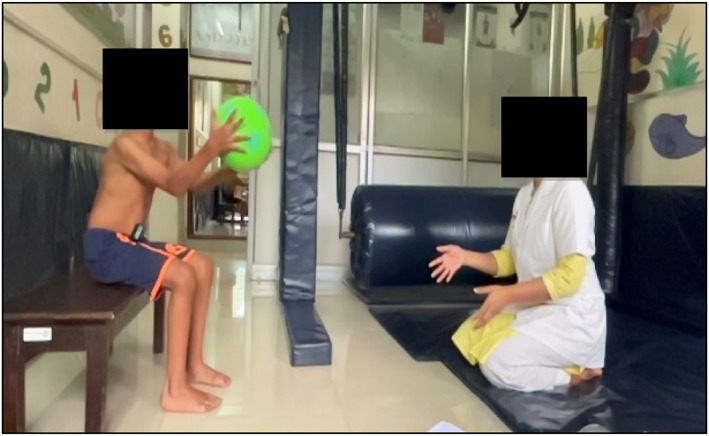
Sitting position.

**FIGURE 2:**
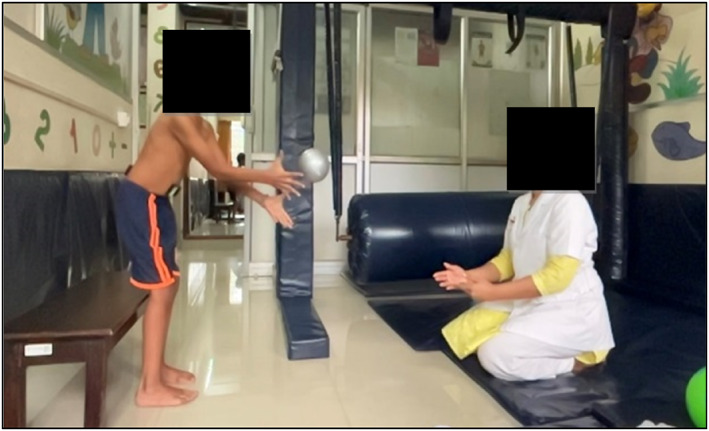
Standing position.

The test was carried out first in a sitting and then in the standing position. The participants were first made to sit on a stool without any back support with hips and knees 90° flexed (Figure 1). They were instructed to catch a ball with both their hands. EMG recording was performed during the activity. Two balls were thrown simultaneously, heavy (1 kg) and light (0.018 kg) from 2 feet. The test was carried out again with both the balls in standing position (Figure 2).

Five practice trials were given in sitting and standing positions to familiarize the children with the activity. Video recording was done to sync the timing of the activities with the EMG data with prior consent. The video camera was placed lateral to the test position of the child at a 4‐foot distance.

#### 2.2.1 Consort Diagram for Subject Selection


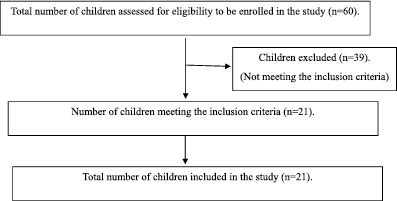


### 2.3 Data Processing

EMG data was obtained using a DataLITE wireless EMG sensor (LE230) with a sampling rate of 500 Hz. The skin was cleaned with alcohol and dried before the electrodes were placed. Using standard adhesive tape, the wireless EMG sensors were applied directly to the skin. The electrodes were placed according to the SENIAM guidelines. The surface EMG signals were collected bilaterally (right and left) from the following muscles: ES—1 cm lateral to the L3 spinous process and RA—3 cm lateral from the umbilicus midline.

EMG data was snipped from 200 ms before the movement initiation for APA, that is, right before the child grasped the ball. For CPA, EMG data was taken 100 ms after initiation of movement. The mean amplitude of compound muscle action potential was recorded for the APA and CPA. The amplitude of muscular activation was calculated during the APA and the CPA for the RA and ES muscles in micro‐volts (μV).

### 2.4 Statistical Analysis

Data distribution was tested for normality using the Shapiro‐Wilk test. Summary information was reported using descriptive statistics (i.e., median and IQR) as the data was not normally distributed. Therefore, non‐parametric tests such as the Wilcoxon sign rank and Mann‐Whitney U tests were used. A non‐parametric test, that is, the Wilcoxon sign rank test, was used to assess the differences in the EMG amplitude during APA and CPA between the two load conditions (light and heavy). Since the EMG amplitude of APA and CPA had significant variation, the difference between APA and CPA in different load conditions and postures was made to verify the relative contribution of CPA compared to APA. Mann‐Whitney *U* test was done to compare the differences in APA and CPA between GMFCS levels I and II in varying load conditions and postures. An alpha level of 0.05 was considered statistically significant.

## 3 Results

The total number of children with CP screened from January 2023 to December 2023 was 60. Only 21 children with CP met the eligibility criteria. Among all the participants, 76.2% (*n* = 16) were males and 23.5% (*n* = 5) were females. According to the gross motor functional characteristics, participants were included in GMFCS levels I and II based on the inclusion criteria. Of the participants, 42.9% (*n* = 9) belonged to GMFCS level I, and 57.1% (*n* = 12) belonged to GMFCS level II. Topographically, 33.3% (*n* = 7) were hemiplegics, and 66.7% (*n* = 14) were diplegics (see Table 1).

**TABLE 1:** Demographic data for children with CP (*N* = 21).

Demographic characteristics	Percentage (%) (number of participants)
Age (5–13 years)	*N* = 21
5–7 years	47.6% (*n* = 10)
7–10 years	33.3% (*n* = 7)
10–13 years	19.1% (*n* = 4)
Gender	
Boys	76.2% (*n* = 16)
Girls	23.5% (*n* = 5)
GMFCS level	
Level I	42.9% (*n* = 9)
Level II	57.1% (*n* = 12)
Diagnosis	
Hemiplegia	33.3% (*n* = 7)
Diplegia	66.7% (*n* = 14)

During APA and CPA, the EMG amplitude was analyzed for two postural muscles, that is, RA and ES, during different load conditions and postures (Table S1).

The APA and CPA were compared between different loads (light and heavy) in sitting and standing (see Table 2).

**TABLE 2:** Comparison of Anticipatory Postural Adjustment (APA) and Compensatory Postural Adjustment (CPA) between two types of loads in sitting and standing positions while grasping a ball in children with CP (*N* = 21).

Postural adjustments	Posture	Type of load	Median (IQR)	*Z*	*p*‐value
			(in μV)		
APA	Sitting	Heavy	0.037 (0.009–0.110)	−1.808	0.07
		Light	0.012 (0.005–0.049)		
	Standing	Heavy	0.017 (0.008–0.048)	−1.409	0.159
		Light	0.018 (0.006–0.056)		
CP	Sitting	Heavy	0.199 (0.129–0.342)	−2.45	0.014*
		Light	0.145 (0.047–0.264)		
	Standing	Heavy	0.204 (0.057–0.287)	−2.381	0.017*
		Light	0.130 (0.029–0.233)		

Abbreviations: IQR, Inter quartile range; μV, micro volt.

*Statistically significant.

Following this, APA and CPA for specific posture and load types were compared to determine the differences in postural adjustments in children with CP (see Table 3).

**TABLE 3:** Comparison of Anticipatory Postural Adjustment (APA) and Compensatory Postural Adjustment (CPA) with respect to type of loads and different postures (*N* = 21).

Posture/Type of load	Postural adjustment	Median (IQR)	*Z*	*p*‐value
		(in μV)		
Sitting/Heavy ball	APA	0.037 (0.009–0.113)	−3.49	0.001**
	CPA	0.199 (0.129–0.342)		
Sitting/Light ball	APA	0.012 (0.005–0.049)	−3.91	0.001**
	CPA	0.145 (0.047–0.264)		
Standing/Heavy ball	APA	0.017 (0.008–0.048)	−3.754	0.001**
	CPA	0.204 (0.057–0.287)		
Standing/Light ball	APA	0.018 (0.006–0.056)	−3.493	0.001**
	CPA	0.130 (0.029–0.233)		

Abbreviation: IQR, Inter quartile range.

**Highly significant at *p* < 0.01 using Wilcoxon Sign Rank test.

Subsequently, based on GMFCS levels, the differences in APA and CPA were analyzed for specific postures and load types (see Table 4).

**TABLE 4:** Comparison of Anticipatory and Compensatory Postural Adjustment for different types of postures and loads between GMFCS level I (*n* = 9) and level II (*n* = 12).

Postural adjustments	Posture/Load	GMFCS	Median (IQR)	Mann Whitney *U* test	*p*‐value
			(in μV)		
APA	Sitting heavy ball	Level 1	0.04 (0.01–0.15)	48	0.118
		Level 2	0.03 (0.01–0.11)		
	Sitting light ball	Level 1	0.012 (0.007–0.03)	38.5	0.35
		Level 2	0.012 (0.012–0.065)		
	Standing heavy ball	Level 1	0.014 (0.006–0.058)	51	0.88
		Level 2	0.027 (0.009–0.05)		
	Standing light ball	Level 1	0.009 (0.005–0.048)	41	0.52
		Level 2	0.019 (0.008–0.065)		
CPA	Sitting heavy ball	Level 1	0.241 (0.093–0.404)	45	0.35
		Level 2	0.16 (0.126–0.288)		
	Sitting light ball	Level 1	0.145 (0.031–0.24)	52	0.83
		Level 2	0.159 (0.049–0.294)		
	Standing heavy ball	Level 1	0.236 (0.052–0.3)	41	0.27
		Level 2	0.181 (0.047–0.288)		
	Standing light ball	Level 1	0.109 (0.023–0.13)	32	0.67
		Level 2	0.16 (0.032–0.34)		

## 4 Discussion

In the present study, the differences in the EMG amplitude for the postural muscles (RA and ES) during APA and CPA between the two load conditions (light and heavy) in children with CP (*N* = 21) were compared in sitting and standing positions. In children with CP, both APA and CPA are affected, hence, the postural control deficits (Hoon et al. 2009). Earlier studies have been conducted on postural control in CP in a sitting position (Van Der Heide et al. 2004; Bigongiari et al. 2011; van der Heide et al. 2003). Some studies included higher‐functioning children with CP; hence, they examined postural control in standing positions (Tomita et al. 2010, 2011; Girolami et al. 2010, 2011). However, to the best of our awareness, no research has been published on postural control in both sitting and standing positions.

The EMG recordings of postural muscles (RA and ES) were analyzed during postural control responses (APA and CPA time epochs). (Table S1).

The postural control responses (APA and CPA) for the heavy and light balls varied in the sitting position. For both APA and CPA, the children with CP showed higher EMG amplitude for RA and ES muscles while grasping the heavy ball; however, for the light ball, it was substantially less (Table 2).

Correspondingly, APA and CPA for the postural muscles (RA and ES) were analyzed with heavy and light balls in the standing position. It was observed that during APA, the amplitude of EMG for the postural muscles remained similar in both the load conditions, that is, heavy and light. Meanwhile, during CPA, the EMG amplitude was higher while catching the heavy ball than during light ball conditions (Table 2).

Additionally, in both sitting and standing positions, the CPA was significantly higher in both ball conditions (heavy and light) than in APA (Table 3).

The available studies on postural responses with additional loads in children with CP showed that for the APA, the percentage intensity of EMG was greater in the trunk muscles for the heavy ball (44.9 ± 20 μV) as compared to the light ball (19.0 ± 9.6 μV). These findings align with the present study specific to the APA. Nevertheless, concerning CPA, the results of this study differed. The likely reason for the same could be the type of movements used to grasp the ball in a seated position, that is, bilateral shoulder flexion (Bigongiari et al. 2011), while in the current study, children with CP had the opportunity of catching the ball with any arm position, in sitting and standing.

The levels of motor disabilities change the characteristics of APA and CPA in children with CP. The current study included higher motor functioning children with CP that is, GMFCS levels I and II. Our findings indicated conflicting patterns of postural control between GMFCS levels I and II in different load conditions and positions (Table 4). The apparent reasons could be the variations in the topographical diagnosis of CP groups, that is, most children in GMFCS level I were spastic hemiplegic, whereas only spastic diplegics were included in level II. Only Tomita H et al. (2015) studied the effects of gross motor disability on APA in a standing position and reported that anticipatory EMG amplitudes of ES were significantly larger for the GMFCS‐II compared to GMFCS‐III (Tomita et al. 2016).

TD children and young adults show changes in postural adjustments with additional load, leading to an increased APA intensity. However, in children with CP, delays in developing specific neural or musculoskeletal subsystems and sensorial deficits contribute to balance difficulties, leading to the inability to modulate APA (Van Der Heide et al. 2004). Nevertheless, in the present study, the children with CP showed considerable change in APA with an added load. This indicated that children with GMFCS levels I and II exhibited near‐normal functions and could engage in more autonomous activities.

A limitation of this study was that kinematic measurements such as COP displacement during anticipatory movement using a force platform were not considered as an outcome measure. Additionally, topographical differentiation was not employed to compare the postural control.

In conclusion, children with CP showed increased EMG amplitude during APA and CPA with a heavier load than light load in sitting and standing positions. Additionally, the EMG amplitude of CPA in sitting and standing for both load conditions was significantly higher than APA. Furthermore, our results showed no relationship between postural control (APA and CPA) and GMFCS levels I and II.

This study's clinical value stems from its ability to enhance postural control in children with CP by focusing on APA and CPA. The study's demonstration of how varying loads impact muscle activation in sitting and standing positions provides beneficial data to structure individualized rehabilitation strategies. Furthermore, employing surface EMG for evaluation provides a tool for tracking the children's progress.

**Implications on physiotherapy practice:** The study's findings suggest that load‐dependent postural training can be incorporated into physical therapy treatment for children with CP. Since postural control mechanisms vary between sitting and standing positions, therapy should include exercises in both postures. This can ensure the comprehensive development of postural adjustments and activation of the postural muscles. Integrating these findings into physiotherapy practice allows therapists to create more effective, individualized intervention programs targeting children with CP's particular postural control requirements, enhancing their functional skills and quality of life.

## Ethics Statement

The scientific and Institutional ethical committee Kasturba Medical College approved the study (IECKMCMLR‐01/2023/18) and met the Ethical principles guidelines of the Declaration of Helsinki. We confirm that we have read the Journal's position on issues involved in ethical publication and affirm that this report is consistent with those guidelines.

## Consent

Informed consent was obtained from all participants and their legal guardians included in the study.

## Conflicts of Interest

The authors declare no conflicts of interest.

## Permission to Reproduce Material From Other Sources

Not applicable.

## Study Registration

Not applicable.

## Supporting information

Table S1

## Data Availability

The data supporting the findings of this study are available upon request. Due to privacy and ethical considerations, the data are not publicly available.
