# Supplementary material for: Anticipatory and Compensatory Postural Adjustments in Sitting and Standing Positions During Functional Activities in Children With Cerebral Palsy
Source: Physiother Res Int. 2025 Jan 13;30(1):e70028. doi: 10.1002/pri.70028 (PMC11727814; doi:10.1002/pri.70028)
Supplement: Supplementary file 1 — Table S1 [file PRI-30-e70028-s001.docx]

| **Postural adjustments** | **Posture/Load** | **Muscles** | **Median (IQR)**  **(in µV)** |
| --- | --- | --- | --- |
| **APA** | Sitting heavy ball | RA | 0.037 (0.006-0.132)  0.021 (0.013-0.053) |
|  |  | ES |  |
|  | sitting light ball | RA | 0.013(0.004-0.055)  0.012 (0.007-0.024) |
|  |  | ES |  |
|  | standing heavy ball | RA | 0.014 (0.007-0.063)  0.017 (0.010-0.063) |
|  |  | ES |  |
|  | standing light ball | RA | 0.008 (0.003-0.021)  0.012 (0.010-0.033) |
|  |  | ES |  |
|  | sitting heavy ball | RA | 0.199 (0.162-0.308)  0.203 (0.101-0.318) |
| **CPA** |  | ES |  |
|  | sitting light ball | RA | 0.181 (0.017-0.256)  0.174 (0.024-0.263) |
|  |  | ES |  |
|  | standing heavy ball | RA | 0.181(0.032-0.309)  0.186 (0.042-0.249) |
|  |  | ES |  |
|  | standing light ball | RA  ES | 0.124 (0.041-0.216)  0.106 (0.023-0.169) |

**Supplemental Document**

**Supplemental table 1: Median EMG amplitude of Rectus abdominis (RA) and Erector Spinae (ES) muscles during APA and CPA in different load conditions and postures while grasping a ball.**
